# Are Anthelminthic Treatments of Captive Ruminants Necessary?

**DOI:** 10.3390/vetsci8100240

**Published:** 2021-10-18

**Authors:** Liron Lahat, Juana M. Ortiz, Paolo Tizzani, Belén Ibáñez, Francisco Valera, Eulalia Moreno, Gerardo Espeso, Rocío Ruiz de Ybáñez

**Affiliations:** 1 Departamento de Sanidad Animal, Facultad de Veterinaria, Campus de Espinardo, Universidad de Murcia, 30100 Murcia, Spain; albeitar88@gmail.com (L.L.); jortiz@um.es (J.M.O.); 2 Dipartimento di Scienze Veterinarie, Università degli Studi di Torino, 10124 Turin, Italy; paolo.tizzani@unito.it; 3 Departamento de Ecología Funcional y Evolutiva, Estación Experimental de Zonas Áridas, Consejo Superior de Investigaciones Científicas, Ctra. de Sacramento s/n, La Cañada de San Urbano, 04120 Almería, Spain; belenibanez@gmail.com (B.I.); pvalera@eeza.csic.es (F.V.); emoreno@eeza.csic.es (E.M.); gespeso@eeza.csic.es (G.E.)

**Keywords:** gazelles, nematodes, anthelmintic treatment, inbreeding, conservation, egg shedding

## Abstract

Anthelmintics are frequently administered to animals to limit fecal egg elimination, so that wild animals in captive breeding programs are treated to maintain a proper health condition. This is effective from a health management perspective, but on the other hand, it could prevent captive animals from developing an effective immunity against parasites that they might encounter when reintroduced into their original geographic areas. The aim of this study was to describe the dynamics of parasite infections in captive Cuvier’s gazelles (*Gazella cuvieri*) not treated with anthelmintics for two years and to evaluate the factors related to their fecal egg shedding. Fifteen one-year-old males were enclosed together and captured monthly to collect feces directly from the rectum. Fecal egg counts were performed, and eggs were classified as strongylid-like, *Nematodirus* sp., or *Trichuris* sp. Fecal egg shedding for the three groups of parasites did not vary significantly over the duration of the study. Only precipitation affected the egg-shedding pattern of all parasites, while inbreeding was positively associated with the number of strongylid-like parasites. These findings suggest an equilibrium between hosts and parasites in absence of treatment during the study. The anthelmintic treatment as a systematic prophylaxis method in captive animals should be avoided and replaced by systematic coprological and clinical vigilance, as well as targeted treatment in the case of a significant rise of fecal egg counts.

## 1. Introduction

Reintroduction programs of endangered species using captive-bred individuals from zoological institutions have been a well-established conservation tool for many years, particularly successful for mammals [1,2]. Ex situ conservation is thus a key tool against extinction in the wild, particularly in the case of species that are currently found only in captivity [3]. There is an ongoing discussion to assess how zoos can contribute in the most effective way to species conservation [4,5], and a proper animal health management within captive breeding programs [6] is a key issue in such a debate.

Anthelmintic drugs are a common tool for parasitic diseases treatment and prophylaxis in zoos [1], and their use was referred to in the guidelines proposed for wildlife prior to translocation and release [7]. In captivity, it is useful only for a short period of time, and animals become reinfected shortly after the interruption of the therapy. High animal density or food and soil contamination due to the difficulty of removing dung from enclosures are some of the factors that may favor reinfections [8]. Therefore, most captive animals harbor a diverse parasite community, and anthelmintic treatments are used only to control and not to eliminate parasitic infections.

High parasitic intensities may cause severe symptoms in hosts: weight loss, weakness, diarrhea, scruffy hair coat, and lethargy [9]. Although permanent parasitism could be a risk for animal health, there is increasing speculation that a low, but constant parasitic burden favors immunity [10], and the International Union for Conservation of Nature (IUCN) considers that it is neither possible, nor desirable for organisms to be “parasite and disease free” [6]. Two main mechanisms contribute to parasite resistance. The first one is the acquired immunity, developed because of a continuous contact with the parasite’s antigens [11]. This contact may enhance host’s response against this parasite and other ones that share the same antigens through the cross-immunity phenomenon [12,13]. The second mechanism depends on the parasite community since interspecific competition for the same niche may prevent new parasites from becoming established [11].

In addition, anthelmintic drug resistance and multidrug resistance (MDR) referring to all three major anthelmintic classes (benzimidazoles, imidothiazoles–tetrahydropyrimidines, and macrocyclic lactones) are a well-documented problem for nematode control in ruminants in many parts of the world (for a review, see [14]). Kaplan [15] described relevant factors affecting resistance acquisition, including the host–parasite relationship, treatment posology, and differences in anthelmintic pharmacokinetics between host species. When dealing with wild animals, and especially with endangered species, whose populations are small, these factors become even more relevant. In these cases, anthelmintic treatment is often irregularly administrated, and host–parasite interactions and drug pharmacokinetics are not always completely understood.

Cuvier’s gazelle *(Gazella cuvieri)* is endemic to Atlas Mountains and neighboring ranges of Northern Africa, from Morocco to Tunisia. It is classified as vulnerable by the IUCN Red List of Threatened Species, with a population estimated at 2360–4560 individuals [16]. This species is managed through the European Endangered Species Programme (EEP), an intensive population management plan for threatened species in zoological institutions of Europe. The EEP is coordinated by the Consejo Superior de Investigaciones Científicas (CSIC) at its facility in Almería, “La Hoya” Experimental Field Station. Although an anthelmintic prophylaxis program is established there, gastrointestinal parasites are frequently detected in periodic fecal exams [17].

The main objective of this study was to explore whether natural processes (e.g., development of acquired immunity, establishment of a parasite community in the hosts) regulate parasite populations in absence of anthelmintic treatments. The working hypothesis is that FEC increases over time in absence of anthelmintic treatments. Alternatively, FEC in absence of treatments will remain relatively constant (due to some of the above-mentioned processes) if the other two components of the epidemiological triad (the host and the environment) are not substantially altered. It is well known that rainfall and temperature affect parasites’ lifecycle and survival of free stages. Here, the influence of those climatic variables on parasite egg shedding was explored. The effect of inbreeding on parasitic infection in gazelles is controversial. Cassinello and others [18] showed that inbreeding affects the parasitic burden of several gazelle species including Cuvier’s gazelle. However, Ibáñez and others [19] found that individual inbreeding had no effect on any of the hematological and serological variables in the same species. In this study, the inbreeding coefficient was also included to clarify this debate.

## 2. Materials and Methods

Study species and location: Cuvier’s gazelle is a small and dark-brown-coated gazelle species. The adult male mean weight is 32.60, whilst females are smaller (mean 26.43). Both sexes have spiraled, fairly straight horns that may reach 39.6 cm in length in males and 29.0 cm in females [20]. A captive population of ca. 150 Cuvier’s gazelles is housed at “La Hoya” Experimental Field Station (La Hoya FS). This captive population descended from four wild-born individuals (one male, three females) arriving at La Hoya FS between 1975 and 1981. La Hoya FS is located in Almería City (36°50′32″ N, 2°28′20″ W), on the southeastern Iberian Peninsula.

The climate in this area is subdesertic, with hot and dry summers and scarce rainfall during the rest of the year. Animals are mainly distributed in reproductive herds of about 5–9 individuals (1 male with 4–8 females and their offspring) or in bachelor groups of males. The enclosure size ranges from 250–500 m^2^. The soil in the enclosures is sandy, and vegetation is very scarce.

Animals are fed daily with fresh alfalfa (frequently, on the ground), commercial pellets, and water. Routine sanitary management of animals includes an annual anthelmintic administration with subcutaneous ivermectin (Ivomec^®^, Boehringer Ingelheim Animal Health, Barcelona, Spain), 0.2 mg/kg of body weight. This treatment is given for the first time when gazelles are 2–6 months old [20]. Fifteen Cuvier’s gazelle males, born between 12 May and 22 June 2009, were removed from their breeding enclosure in May 2010 and located in a fence that had not been in use for a year to establish the study group. Since the purpose of La Hoya FS is the conservation of endangered species, the experimental design had to be adapted to the management needs, so that only a study group with males could be formed for a two-year study. As in the rest of the cases, the soil of the enclosure was mainly sandy, with no grass. The density of animals remained unchanged throughout the study, with no rotation of the soil with other species or with other individuals of the same species. A clinical evaluation of the animals was carried out every morning, noting their general condition, the consistency of the feces eliminated, and the overall feed consumption. A previous study [17] described nine species of abomasal and small intestinal nematodes parasitizing Cuvier’s gazelle in La Hoya FS: *Camelostrongylus mentulatus*, *Nematodirus filicollis*, *N. helvetianus*, *N. spathiger*, *Ostertagia harrisi*, *O. ostertagi*, *Teladorsagia* (*O*.) *circumcincta*, *T.* (*O*.) *davtiani*, *Trichostrongylus colubriformis*, *T. probolurus*, and *T. vitrinus* [17]. For some of these parasites, resistance mechanisms such as hypobiosis have been described [21,22,23,24]. Animals included in the present study had not received anthelmintic treatment at all, even before their movement to begin the experiment.

Determination of the egg-shedding pattern: sampling procedure and parasitological analyses: Between May 2010 and April 2012, the 15 individuals were captured monthly, using a net system [20], which enables harmless confinement in accordance with the Spanish regulation RD 1201/2005 and European Union Regulation 2003/65/CE. Immediately after capture, their legs were tied, and their faces masked to reduce stress [25]. Fecal samples were collected from the rectum (166 samples during 2010/2011 and 145 along 2011/2012; 21 samples/animal ± 4.32, minimum–maximum value: 10–24, n = 15) and individually stored. The samples were sent to the Animal Health Department of the University of Murcia and conserved at −20° for further analyses. Fecal samples were analyzed using a modified McMaster technique [26].

Feces (3g) were homogenized with tap water, filtered, and centrifuged (650× *g*, 5 min). The sediment was thoroughly mixed with Sheather’s solution (specific gravity = 1.27), and McMaster chamber slides were filled in triplicate for fecal egg counts. The sensitivity of the test was 15 eggs/g. Based on morphometry [27], the eggs were categorized into three groups (strongylid-like, *Nematodirus* sp., or *Trichuris* sp.).

Host- and environment-related factors: The captive population of Cuvier’s gazelle is highly inbred due to its low number of founders (1 male, 3 females). Considering the likely effect of the inbreeding coefficient on parasite burden [18], we included this variable as a potential predictor of egg shedding. Individual inbreeding coefficients were calculated using the SPARKS software program [28].

To evaluate the influence of climatic variables on the egg-shedding pattern, precipitation (mm), temperature (°C), and solar radiation data (kW/m^2^) (mean by month) from May 2010 to April 2012 were obtained from a meteorological station: (36°50′47″ N. 2°21′24″ W) 11.5 km away from La Hoya FS. These three factors are well known to exert a critical influence on the survival of parasites [29].

Statistical analysis: Egg shedding was studied based on the number of eggs/g of feces for each individual. Monthly median values and their range (difference between the lowest and the highest value) were also calculated. The normality of the variables was evaluated using the Shapiro–Wilk test. The nonparametric Friedman chi-squared test for repeated measures was used to look for yearly and monthly differences in the median number of eggs/g of feces for each parasite group. Generalized Linear Mixed Models (GLMM) were used to evaluate the relation between the parasites’ group egg shedding and extrinsic (precipitation, temperature, solar radiation, month, year) or intrinsic factors (inbreeding). Individual effects were used as random factors. Models with the lowest value of Akaike’s Information Criterion (AIC), indicating a better model fit, were selected. Statistical significance was set at *p* < 0.05 for all analyses. Statistical analyses were conducted using R software [30].

## 3. Results

### 3.1. Prevalence and Abundance of Gastrointestinal Parasites

All individuals were parasitized by the three parasitic groups considered in this study. Gastrointestinal (GI) nematode eggs were detected in 310 out of 311 samples (99.7%). However, the daily clinical follow-up of the animals in the study group did not reveal any symptoms that could be related to parasitic gastroenteritis. Median monthly excretion of *Trichuris* eggs was significantly higher than that for strongylid-like and *Nematodirus* eggs (Friedman chi-squared = 13.62, *p* = 0.001) (Table 1).

### 3.2. Temporal Pattern of Egg Shedding and Interindividual Variability

The prevalence and egg shedding of studied animals during the whole study period (expressed in terms of eggs per gram of feces) are summarized in Table 2.

**Figure 1:**
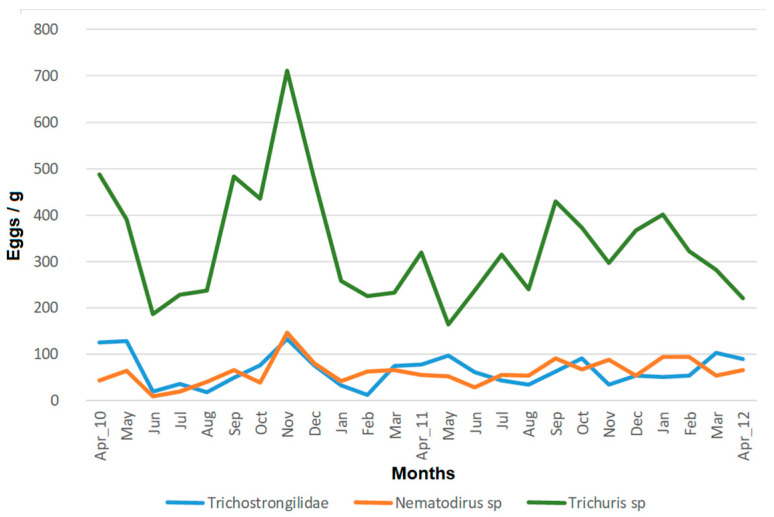
Median monthly fecal egg counts (eggs/g) of three nematode groups from Cuvier’s gazelles between April 2010 and 2012.

Significant monthly fluctuations in the median number of eggs/g were found for all the GI parasites (Friedman chi-squared = 292.29, *p* < 0.001 for strongylid-like eggs, Friedman chi-squared = 316.16, *p* < 0.001 for *Nematodirus* eggs, and Friedman chi-squared = 557.49, *p* < 0.001 for *Trichuris* eggs) (Figure 1). During the first year, the three parasitic groups showed the maximum egg shedding in November. However, the dynamics of excretion were not so coincident in the second year, and the maximum seemed to be ahead of the first year of study. No differences in the number of eggs/g were found between the first and the second year for any of the GI parasites (Friedman chi-squared = 0.005, *p* > 0.1 for strongylid-like eggs, Friedman chi-squared = 4.033 *p* > 0.1 for *Nematodirus* eggs, and Friedman chi-squared = 5.078, *p* > 0.1 for *Trichuris* eggs).

Finally, the median values of egg excretion showed wide differences among individuals (Coefficient of Variation (CV) strongylid-like = 121.30%, CV *Nematodirus* = 106.05%, CV *Trichuris* = 121.69%).

### 3.3. Factors Influencing the Egg Shedding Pattern

The multivariate analysis (GLMM with a Gaussian distribution and the identity link function) revealed a significant and positive effect of precipitation on the egg-shedding pattern of all the parasitic groups (*p* < 0.01; Table 3). Neither environmental factors such as ambient temperature and solar radiation, nor factors related to sampling temporality such as the year or month of sample collection had any effect on egg-shedding patterns. In addition, the inbreeding coefficient was positively correlated with the number of strongylid-like parasites found, but not with the other parasites (Table 3).

## 4. Discussion

This study was carried out with male Cuvier’s gazelles, which had not received anthelmintic treatment at all. The results showed that almost all of them (99.7%) shed nematode eggs. The constant presence of parasites may be due to (i) continuous infection from the contaminated environment [8,31] and (ii) the hypobiosis phenomenon described for some gastrointestinal nematode species such as *O. ostertagi* and *C. mentulatus* [21,22,23,24].

Despite the deworming protocol followed in La Hoya FS, newborn gazelles are regularly infected. This fact could be attributable to several reasons (for a review, see [14]), including the lack of knowledge of the pharmacokinetics and pharmacodynamics of most drugs used in wildlife [32], overuse of a specific anthelmintic and underdosing [33], development of nematode resistance to anthelmintics [34], or confinement of animals in the same areas [35].

All gastrointestinal nematodes’ eggs recovered over the study period showed baseline levels throughout most of each year, with a tendency to drop during the hot and dry summer season and from December to February, although the differences between seasons were not statistically significant. Only in October/November 2010, a peak of fecal egg shedding was recorded; however, a similar peak was not confirmed during the same period the following year. These may explain the lack of statistical significance found between both years’ results. A similar twelve-month study conducted in La Hoya FS by Ortiz and others [24] in the same species’ breeding herds revealed a similar egg-excretion pattern. Those authors suggested the existence of FEC peaks related to periparturient females. The results from this study showed the same pattern in bachelor males, which suggests that the egg-shedding pattern found is common at the species level, irrespective of sex, age, or breeding status of the individual, being influenced only by precipitation in this study.

In this sense, harsh environmental conditions during the hot and dry summer in semiarid areas such as the southeast of Spain can compromise strongylid-like eggs’ viability and larval survival on pasture and favor either hypobiosis in the abomasal mucosa or survival as low-fecundity adults. The development of the two first larval stages inside *Nematodirus* eggs seems to protect them from these extreme conditions [24]. On the other hand, the relatively high egg shedding of *Trichuris* spp. over the period of study is remarkable. This nematode could evade therapeutic interventions and persists in hosts due to the resistance of the eggs to adverse environmental conditions and due to the long lifespan of adult worms [36].

Although short-term oscillations in excretion were detected during the study period, egg-shedding levels, instead of increasing, stabilized, showing no significant differences along the two years of the study. This result suggests that a host–parasite equilibrium might be developed in La Hoya FS’s captivity conditions, even if anthelmintic treatment is not administered. This equilibrium prevents FEC from reaching high levels of infection as evidenced by the fact that no clinical disease was detected in the untreated Cuvier’s gazelles during the whole study period. In this sense, only short-term FEC exceeded 100 epg, a limit value for ruminant gastrointestinal nematodes not to cause clinical signs [8]. In the case of *Trichuris*, fecal egg counts were always less than 1500 epg, a value considered moderate for this parasite [37].

This result suggests the possibility of limiting anthelmintic treatments to certain specific situations, particularly those that cause a high level of stress to the animals. Concern about the use of anthelmintics in these animal populations have led to the search for alternative solutions [38]. According to other authors, many benefits reside in the reduction of the usage of anthelmintics such as a regular prophylactic treatment. The most important one is that the population could develop a specific immunity against these parasites. If treated gazelles are reintroduced to their original range, they would probably encounter parasites at the same time as other stressors (transport, new husbandry, contact with other animal groups, different climatic conditions, and nutrition, etc.), which would be able to cause immunosuppression [10]. Then, parasites could proliferate, causing morbidity and even mortality in animals. For example, three dorcas gazelles (*Gazella dorcas neglecta*) from La Hoya FS that were relocated to a zoo in Berlin died of abomasitis caused by *C. mentulatus* [39], the most prevalent trichostrongylid found in La Hoya FS [17]. However, if animals are not routinely dewormed, they should develop sufficient immunity, so the stressors associated with relocation do not result in massive multiplication of nematodes. Another profit of limiting anthelmintics is the possibility to detect individuals highly susceptible to parasite infections, which may not be adequate candidates for reproduction programs. Additionally, an important advantage of the proposed measure is the reduction of unnecessary manipulations of gazelles and, finally, preventing anthelmintic drug resistance development.

On the other hand, certain anthelmintics might be ecotoxic to organisms enriching the soil. In this sense, Verdú and others concluded that massive treatment of entire flocks in a given area can lead to a significant reduction in the number and composition of coprophagous invertebrates, delaying dung and altering soil nutrient cycling [40,41]. Furthermore, Schulte-Hostedde and others [42] demonstrated that one consequence of treatment is that the immune systems of captive populations do not have the adequate pathogens to fend off and then become dysfunctional. In this sense, the anti-inflammatory properties of helminth worms are now recognized, and the therapeutic use of selected species against inflammatory bowel disease is being tested [43], explaining inflammation as a result of the impaired functionality of the immune system that is traditionally adapted to fight against worms, which it does not encounter now [44]. Captive populations may be at particular risk of this chronic inflammation because of the interaction between the immune system and stressful situations [45].

The GLMM analysis revealed that precipitation had a positive association with the egg shedding of all studied parasitic groups and inbreeding positively affected only the strongylid-like egg-shedding pattern. Previous studies provided strong evidence that precipitation plays a significant role in larval migration from soil to vegetation under both field and laboratory conditions (reviewed in [46]) and, therefore, in their ability to infect new hosts. Temperature and direct sunlight are two other factors with recognized importance in the development of parasites [47]. However, none of the latter proved to have a significant influence in our study. The gazelles housed in La Hoya FS do not graze, as would occur in natural conditions, but are fed mostly in roof-covered feeders. These conditions probably reduce the influence of solar radiation and even temperature. However, the eventual existence of a microclimate in La Hoya FS, with some characteristics slightly different from those collected at the weather station 11.5 km away from the study area, should be considered in the data interpretation.

The effect of inbreeding on parasite populations has been related to the evidence that inbreeding decreases fitness in captive animals (reviewed in [48]), including Cuvier’s gazelle [18]. However, in this study, the inbreeding coefficient only affected the egg-shedding pattern of strongylid-like parasites. This result seems to be more in agreement with Ibáñez and others [19], who did not find any effect of this variable on any of the hematological and serological variables related to health status that they studied. The detrimental effect of inbreeding is generally assumed, as well as its negative impact on fitness, including parasite burden [18]. However, it has been demonstrated that in this captive Cuvier’s gazelle population, a non-negligible phenotypic variation for a key fitness trait such as juvenile survival is ascribed to additive genetic variance (*h*^2^, heritability). Although further studies would be necessary to confirm this, it may be that, similarly to juvenile survival, in the captive population of Cuvier’s gazelle studied here, there is a considerable additive genetic variance for resistance to *Trichuris* and *Nematodirus* sp. parasites, as that previously found in Scotland for parasite resistance in Soay sheep (*Ovis aries*) measured as fecal egg counts and body size [49]. This is also in agreement with the findings by Mitchel and others [50], who demonstrated that heterozygosity rather than inbreeding predicts parasite burden in wild banded mongooses (*Mungos mungo*). The different pattern found in strongylid-like egg shedding is intriguing. Local genetic effects, the linkage between genetic markers and genes influencing parasite burdens, might explain it [50], although it cannot be ruled out that it could be a random effect.

Our results suggest that anthelmintic treatment as a systematic prophylaxis method could be avoided for captive animals since gazelles remain healthy, without clinical signs of gastrointestinal damage (diarrhea, anorexia, weight loss, etc.), at least along the two years of study. This practice could be replaced by a systematic coprological and clinical vigilance to perform targeted treatments, mainly when animals are proposed to be relocated. We propose that precipitation and inbreeding (for some parasites) could determine the time points at which to administer anthelmintics, if necessary, in the absence of stressful conditions.

Zoos continue to face challenges in maintaining populations of endangered species in captivity while integrating evolutionary perspectives into management protocols that may enhance success. New approaches to minimize the development and presence of nematodes in the soil [5,51,52], and so the administration of anthelmintics, are needed. In order to ensure the self-regulation of nematodes’ egg shedding in captive ruminants, new research should be conducted over a longer period, including animals located in different enclosures and of different age and sex classes (e.g., adults, senior, periparturient females, and young nimals).

## Figures and Tables

**Table 1:** Prevalence and fecal egg count of the three parasite groups during the study period (May 2010–April 2012). n = 15 individuals.

Species	Prevalence	Minimum Median	Maximum Median	Overall Median	Overall Q1	Overall Q2
Strongylid-like	100%	0 ^1^	118.5	42.3	13.15	87
*Nematodirus*	100%	6.35	140.4	40.4	13.9	81.8
*Trichuris*	100%	97.3	573.5	208.3	118.3	412.2

^1^ Number of eggs per gram of feces.

**Table 2:** Egg-shedding pattern of each group of parasites over the study period (May 2010–April 2012) for each Cuvier’s gazelle. Prevalence refers to the number of positive samples collected for each animal (number of samples in brackets).

	Strongylid-like			*Nematodirus*			*Trichuris*		
Animal ID	Prevalence	Median ^1^	Range ^2^	Prevalence	Median	Range	Prevalence	Median	Range
42	71% (24)	14.4	168.2	71% (24)	28.6	155.6	100% (24)	118.2	726.3
45	91% (22)	48.6	228.6	50% (22)	6.35	122.2	95% (22)	210.6	733.5
47	90% (21)	118.5	472.4	95% (21)	140.4	493.8	100% (21)	166.7	1406.5
48	70% (20)	51.1	246.9	90% (20)	59.2	193.2	100% (20)	322.8	1481.5
50	83% (24)	42.7	257.2	54% (24)	13.9	44.4	100% (24)	260.9	666.1
51	85% (13)	44.2	223.7	58% (13)	14.7	89.5	92% (13)	227.5	1804.6
52	70% (10)	24.2	310.9	50% (10)	7.15	74.1	100% (10)	97.3	1940.8
53	94% (16)	107.1	429.6	81% (16)	43.7	255.3	100% (16)	492.1	1128.0
54	91% (23)	58.3	308.0	91% (23)	29.6	239.7	100% (23)	184.1	1436.8
56	71% (24)	2.9	277.8	88% (24)	40.4	277.8	100% (24)	246.6	800.5
60	41% (22)	0.0	80.3	73% (22)	47.0	167.5	91% (22)	118.1	318.6
61	61% (23)	2.9	173.0	87% (23)	44.0	213.3	100% (23)	332.6	1248.3
62	82% (22)	44.7	244.4	82% (22)	44.4	132.9	100% (22)	233.2	1352.6
65	61% (23)	28.7	241.7	91% (23)	88.9	555.6	100% (23)	573.5	1859.3
67	79% (24)	31.2	103.4	78% (24)	43.6	192.0	100% (24)	133.3	518.7

^1^ Median monthly number of eggs per gram of feces. ^2^ Range of the median values.

**Table 3:** Selected models for the three parasite groups. Estimates, standard errors, degrees of freedom (df), *t*-values, and *p*-values are displayed.

Strongylid-like Eggs—AIC = 3547 − Marginal R2 = 0.09/Conditional R2 = 0.19					
	Estimate	Std. Error	df	*t* value	Pr(>|t|)
(Intercept) *	−265.962	114.965	11.877	−2.313	0.039
Precipitation	0.513	0.197	297.148	2.610	0.010
inbreeding	1399.020	498.618	11.888	2.806	0.016
*Nematodirus*—AIC = 3469 − Marginal R2 = 0.11/Conditional R2 = 0.34					
	Estimate	Std. Error	df	*t* value	Pr(>|t|)
(Intercept)	−255.723	157.156	12.555	−1.627	0.129
precipitation	0.769	0.177	294.397	4.344	0.000
inbreeding	1309.332	681.496	12.557	1.921	0.078
*Trichuris*—AIC = 4463 − Marginal R2 = 0.03/Conditional R2 = 0.20					
	Estimate	Std. Error	df	*t* value	Pr(>|t|)
(Intercept)	172.3653	618.7681	12.8382	0.2790	0.7850
precipitation	2.6999	0.8483	297.0684	3.1830	0.0016
inbreeding	506.9516	2683.4574	12.8453	0.1890	0.8531

* The intercept (often labelled the constant) is the expected mean value of Y when all X = 0.

## Data Availability

The data presented in this study are available on request from the corresponding author.
